# Anxiety and Suicidal Thoughts During the COVID-19 Pandemic: Cross-Country Comparative Study Among Indonesian, Taiwanese, and Thai University Students

**DOI:** 10.2196/24487

**Published:** 2020-12-24

**Authors:** Iqbal Pramukti, Carol Strong, Yajai Sitthimongkol, Agus Setiawan, Moses Glorino Rumambo Pandin, Cheng-Fang Yen, Chung-Ying Lin, Mark D Griffiths, Nai-Ying Ko

**Affiliations:** 1 Faculty of Nursing Universitas Padjadjaran West Java Indonesia; 2 International Doctoral Program in Nursing, Department of Nursing College of Medicine National Cheng Kung University Tainan Taiwan; 3 Department of Public Health College of Medicine National Cheng Kung University Tainan Taiwan; 4 Faculty of Nursing Mahidol University Bangkok Thailand; 5 Faculty of Nursing Universitas Indonesia Jakarta Indonesia; 6 Faculty of Humanities Universitas Airlangga East Java Indonesia; 7 Department of Psychiatry Kaohsiung Medical University Hospital & School of Medicine College of Medicine Kaohsiung Medical University Kaohsiung Taiwan; 8 Institute of Allied Health Sciences, National Cheng Kung University Hospital College of Medicine National Cheng Kung University Tainan Taiwan; 9 International Gaming Research Unit Psychology Department Nottingham Trent University Nottingham United Kingdom; 10 Department of Nursing, National Cheng Kung University Hospital College of Medicine National Cheng Kung University Tainan Taiwan

**Keywords:** anxiety, COVID-19, cross-country, suicidal thoughts, university students

## Abstract

**Background:**

The COVID-19 pandemic has negatively affected the mental health of university students.

**Objective:**

This study examined the psychological responses toward COVID-19 among university students from 3 countries—Indonesia, Taiwan, and Thailand.

**Methods:**

We used a web-based, cross-sectional survey to recruit 1985 university students from 5 public universities (2 in Indonesia, 1 in Thailand, and 1 in Taiwan) via popular social media platforms such as Facebook, LINE, WhatsApp, and broadcast. All students (n=938 in Indonesia, n=734 in Thailand, and n=313 in Taiwan) answered questions concerning their anxiety, suicidal thoughts (or sadness), confidence in pandemic control, risk perception of susceptibility to infection, perceived support, resources for fighting infection, and sources of information in the context of the COVID-19 pandemic.

**Results:**

Among the 3 student groups, Thai students had the highest levels of anxiety but the lowest levels of confidence in pandemic control and available resources for fighting COVID-19. Factors associated with higher anxiety differed across countries. Less perceived satisfactory support was associated with more suicidal thoughts among Indonesian students. On the other hand, Taiwanese students were more negatively affected by information gathered from the internet and from medical staff than were Indonesian or Thai students.

**Conclusions:**

Our findings suggest that health care providers in Thailand may need to pay special attention to Thai university students given that high levels of anxiety were observed in this study population. In addition, health care providers should establish a good support system for university students, as the results of this study indicate a negative association between support and suicidal thoughts.

## Introduction

The COVID-19 outbreak was declared as a pandemic on March 11, 2020, by the World Health Organization [1]. It has affected over 100 countries worldwide, including many countries in the Asia-Pacific region. As of October 20, 2020, nearly 40 million confirmed cases of COVID-19 were reported globally, with over 9 million cases reported in the Asia-Pacific region [1]. In Indonesia, Thailand, and Taiwan, in particular, the total number of confirmed COVID-19 cases was over 137,000; 3300; and 480, respectively, as of August 16, 2020 [1-3].

With increasing numbers of COVID-19 infections and associated deaths, psychological responses toward COVID-19 have become an important topic for health care providers [4,5]. Indeed, studies on different populations (including pregnant women and their husbands, general population, and children) have reported increased psychological problems during the COVID-19 pandemic [6-8]. Moreover, instruments assessing psychological responses specifically designed in relation to COVID-19 have been developed and validated [9-14]. Therefore, the importance of assessing psychological responses toward COVID-19 and their associated factors has been highlighted in the extant literature. More specifically, adverse effects of elevated psychological distress may trigger reassurance-seeking and compulsive-checking behaviors in response to potential threats of COVID-19 infection, which, in turn, may have an impact on the daily lives of individuals who impede preventive behaviors [15,16].

Although university students worldwide are affected by the COVID-19 pandemic, their psychological responses may be different because of cultural differences and varying levels of the COVID-19 crisis across countries. For example, Taiwan undertook early action to prevent COVID-19 transmission and maintained an almost normal living state without having to implement a national lockdown [10,15,17]. Such a situation may have mitigated negative psychological responses among Taiwanese university students. On the other hand, COVID-19–related fatality rate in Indonesia was found to be relatively high (8.9%), and it may have more negatively affected university students than other countries [18].

This study examined university students’ psychological responses toward COVID-19 for the following reasons. First, the COVID-19 pandemic has become a major life stressor that has direct and indirect influences on students’ psychological well-being. The direct influences include the students’ emotional feelings toward COVID-19 (eg, fear of being infected) [19-21], and the indirect influences include the government’s reaction in relation to transmission control (eg, feeling depressed when receiving threatening COVID-19 news or feeling lonely because of social distancing) [7]. Second, university students are at a critical life-transition period (ie, school-to-work transition [22]). Most of them are expected to begin their careers after graduation by applying the skills they have learned at the university [23]. However, the COVID-19 pandemic may interfere with their plans and, subsequently, affect their future careers. Therefore, university students need support in maintaining good mental health conditions in order to deal with the COVID-19 pandemic, as well as avoid any negative consequences later in life.

Therefore, in this study, we applied a combination of the health belief model (HBM) [24] and protection motivation theory (PMT) [25], to examine potential independent variables for explaining psychological responses among university students. The HBM posits that perceived susceptibility, perceived severity, and perceived benefits are the major contributors enabling individuals to take specific health behavioral actions. Moreover, the PMT posits that individuals’ health-related behaviors are triggered by their psychological distress (eg, anxiety and fear). Therefore, the factors proposed by the HBM are potential independent variables that help explain the psychological responses of individuals. In this study, we assessed the factors proposed by the HBM as follows: perceived susceptibility and perceived severity were assessed using risk perception of susceptibility to COVID-19; perceived benefits were assessed using confidence in pandemic control (ie, controlling the pandemic is a benefit for the individual to fight COVID-19), perceived support (ie, having support is a benefit for the individual to fight COVID-19), and perceived sufficiency of resources (ie, having sufficient resources is a benefit for the individual to fight COVID-19).

In addition to applying the HBM [24] and PMT [25], the existing literature on COVID-19 also suggests that these independent variables could contribute to an individual’s psychological responses. For instance, a higher confidence in fighting COVID-19 may be associated with more adaptive psychological responses when dealing with COVID-19 [17,26]. Fear of COVID-19 was found to be lower when the country had better control of COVID-19 cases. As people are known to have fear of COVID-19 [27] and being stigmatized if they are infected [28], they may have more negative psychological responses when they perceive higher risk of having COVID-19. In addition, previous studies have made recommendations to provide sufficient resources such as personal protective equipment (PPE) and support to assist individuals in combating psychological problems during the COVID-19 outbreak [29,30].

Apart from the HBM and PMT, the literature proposes the importance of obtaining accurate COVID-19 information from trusted sources. Indeed, misinformation concerning COVID-19 is associated with a greater fear of the disease [15,16]. Hou et al [31] reported that rumors and misinformation shared on the internet may induce panic behaviors such as purchasing herbal remedies via web-based shopping and storing them. Based on the HBM, PMT, and the existing literature, we hypothesized that confidence in COVID-19 pandemic control, risk perception of susceptibility to COVID-19, perceived satisfactory support, perceived sufficiency of resources for fighting COVID-19, and sources of obtaining COVID-19 information are all potential factors that may be associated with psychological responses to COVID-19.

To the best of our knowledge, there have been few cross-country comparisons concerning psychological responses among university students. In order address this gap in the literature, we compared the psychological responses toward COVID-19 and its related factors among university students in 3 different countries—Indonesia, Thailand, and Taiwan. These 3 countries were selected for the comparative study because they are all East Asian countries that share similar cultures and values (ie, Confucianism) [32]. In addition, a previous study on country variations concerning depression symptoms found similar prevalence of low self-confidence in these 3 countries [33]. However, the 3 countries had different outcomes and policies in minimizing the impact of the COVID-19 pandemic. Therefore, it would be useful to apply the same model to 3 countries that have similar cultures and values but had implemented different measures in combating COVID-19.

The primary outcomes of this study were different psychological responses such as anxiety and suicidal thoughts. Moreover, we examined other related factors (ie, confidence in COVID-19 pandemic control, risk perception of susceptibility to COVID-19, perceived satisfactory support, perceived sufficiency of resources for fighting COVID-19, and sources of obtaining COVID-19 information) to understand their associations with psychological responses among different university student groups from different countries.

## Methods

### Study Design, Participants, and Data Collection

A multicountry, web-based cross-sectional study was conducted in 5 public universities. The sample comprised students in Indonesia (2 universities), Taiwan (1 university), and Thailand (1 university). Participants were recruited through popular social media platforms operational in these countries, including Facebook, LINE, WhatsApp, and Broadcast, from April 10 to June 30, 2020. Only participants aged 20 years and above were eligible for this study. Before beginning the survey, the participants were asked to log in with their personal email addresses in order to avoid having participants repeat the survey more than once.

We obtained approvals from the Research Ethics Committee in each of the 3 countries studied (ie, University of Indonesia [SK-139/UN2.F12.D1.2.1/ETIK 2020] for Indonesia, National Cheng Kung University Hospital [A-EX-109-019] for Taiwan, and Mahidol University [COA No. MU-COVID 2020.006/1205] for Thailand). Thereafter, data collection was initiated. Participation in the survey was voluntary, and survey responses were anonymously collected. Study participants were given no incentive for participation. Participants who agreed to participate in the study completed the web-based survey in their native languages: Bahasa (Indonesian students), Mandarin (Taiwanese students), and Thai (Thai students).

### Measures

#### Outcome Variables: Anxiety and Suicidal Thoughts (or Sadness)

The State-Trait Anxiety Inventory (STAI) was used to assess the anxiety levels of the participants toward the COVID-19 pandemic. The STAI comprises 10 items rated on a 4-point Likert scale (“not at all,” “a little,” “somewhat,” and “very much”). A lower score on the STAI indicates a lower level of anxiety [34]. Suicidal thoughts in the past week were assessed for Taiwanese and Indonesian students on a 5-point Likert scale (“not at all,” “mild,” “moderate,” “severe,” and “very severe”). A lower score on this scale indicates a lower level of suicidal ideation. Similarly, sadness experienced in the past week was assessed for Thai students on the same 5-point Likert scale. Although sadness is not a direct concept of suicidal thoughts, it can be viewed as a proxy of suicidal thoughts for Thai students. More specifically, we considered that when a Thai student feels sad for a prolonged period, their physiological and psychological functions will be disturbed and may further lead to depressive symptoms with a high risk of suicidal ideation.

#### Independent Variables

##### Confidence in Pandemic Control

Confidence in pandemic control included confidence concerning personal, city, and the university in handling the pandemic situation. This variable was rated on a 5-point Likert scale from 1 (“not confident at all”) to 5 (“very confident”) [35]. The Cronbach alpha of the 3 items concerning confidence in pandemic control indicated very good internal reliability (Cronbach α=.83).

##### Risk Perception of Susceptibility to COVID-19

Risk perception of susceptibility to COVID-19 included both absolute susceptibility and relative susceptibility, both of which were rated on a 7-point Likert scale from 1 (“not at all susceptible”) to 7 (“very susceptible”) [36]. The Cronbach alpha value of the 2 items concerning risk perception of susceptibility to COVID-19 indicated very good internal reliability (Cronbach α=.80).

##### Perceived Support

Perceived support assesses support received from families, classmates, and faculties. The 3 items were rated on a 5-point Likert scale from 1 (“not satisfied at all”) to 5 (“very satisfied”) [37]. The Cronbach alpha value of the 3 items concerning perceived support indicated acceptable internal reliability (Cronbach α=.69).

##### Perceived Sufficiency of Resources for Fighting COVID-19

Perceived sufficiency of resources included perceived sufficiency of PPE, COVID-19 information, money, medical resources, and psychological support. All items were rated on a 3-point Likert scale from 1 (“insufficient”) to 3 (“sufficient”) [38]. The Cronbach alpha value of the 5 items concerning perceived sufficiency of resources for fight COVID-19 indicated very good internal reliability (Cronbach α=.76).

##### Sources of COVID-19 Information

Seeking information from various sources included information from the internet, medical staff, and family [39]. All items were rated on a 3-point Likert scale from 1 (“never”) to 3 (“always”).

#### Demographic Information

Sociodemographic data such as gender, age, and education level were collected from the survey responses. Students with a Bachelor’s qualification were classified as undergraduates, and those with a Master’s qualification or above were classified as postgraduates. Students were also asked whether their major subjects of study were health-related or not.

### Statistical Analysis

**Figure 1:**
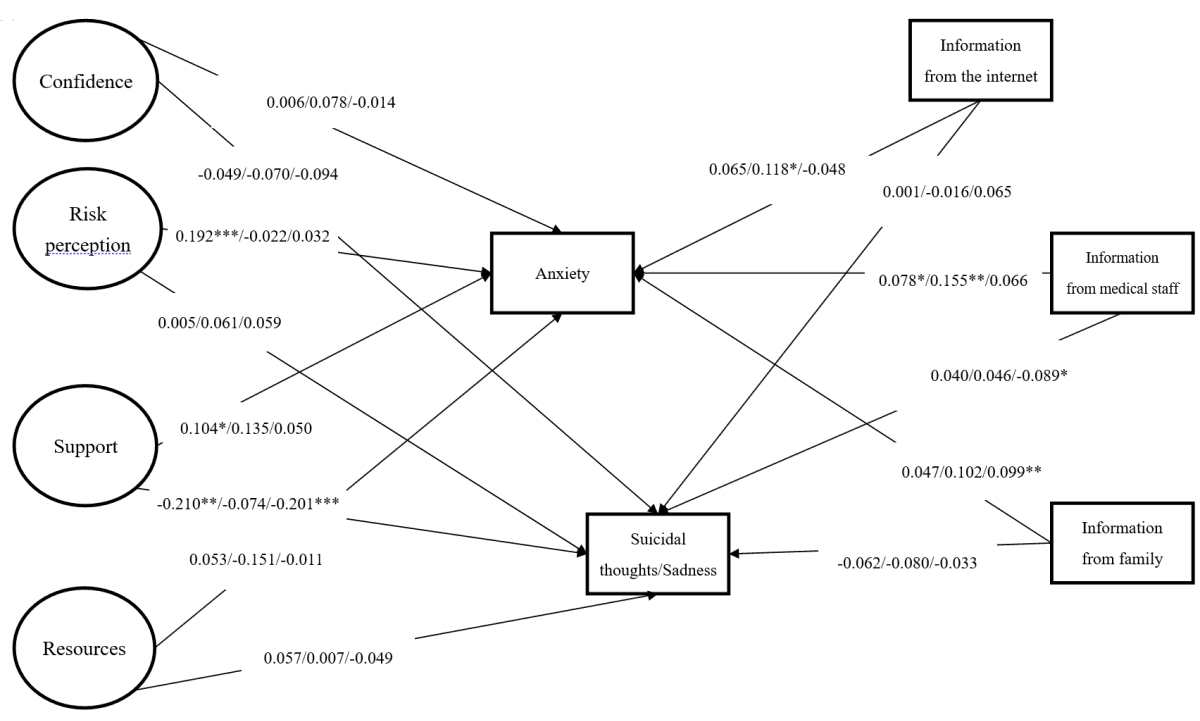
Results of the proposed model showing standardized coefficients among Indonesian, Taiwanese, and Thai students. *P<.05, **P<.01, ***P<.001. Gender was controlled for in the model. Suicidal thoughts were assessed for Taiwanese and Indonesian students; sadness was assessed for Thai students. Confidence was constructed using (1) confidence to deal with the pandemic, (2) confidence of the city to deal with the pandemic, and (3) confidence of students to deal with the pandemic. Risk perception was constructed using (1) perceived absolute susceptibility to COVID-19 and (2) perceived relative susceptibility to COVID-19. Support was constructed using (1) satisfaction with family support, (2) satisfaction with friend support, and (3) satisfaction with university support. Resource was constructed using (1) sufficiency of personal protective equipment, (2) sufficiency of information, (3) sufficiency of money, (4) sufficiency of medical resources, and (5) sufficiency of psychological support. Fit indices: χ2378=1035.12; *P*<.001; comparative fit index=0.92; Tucker-Lewis index=0.90; root mean square error of approximation=0.051 (90% CI 0.048-0.055); standardized root mean square residual index=0.053.

Analyses of variance (ANOVA) and *χ^2^* tests were used to examine the differences concerning dependent variables, independent variables, and controlled variables. Posthoc comparisons with Bonferroni adjustments were used when an overall statistical significance was observed in ANOVAs or χ^2^ tests. Pearson correlations were then used to understand the bivariate correlations between the studied variables for all participants. Multigroup structural equation modeling (SEM) was then applied to examine how the independent variables explained anxiety and suicidal thoughts (for Taiwanese and Indonesian students) or anxiety and sadness (for Thai students; see Figure 1). In the multigroup SEM, confidence in fighting COVID-19, risk perception of susceptibility to COVID-19, perceived satisfactory support, and perceived sufficiency of resources were latent variables, whereas anxiety, suicidal thoughts (or sadness), sources of COVID-19 information (internet, medical staff, and family), and gender were manifest variables. Weighted least squares mean and variance adjusted estimator was used in the multigroup SEM to address the nature of Likert-type scales used in the study measures. Fit indices such as comparative fit index (CFI), Tucker-Lewis index (TLI), root mean square error of approximation (RMSEA), and standardized root mean square residual (SRMR) were used to determine whether the multigroup SEM is supported (CFI and TLI should be 0.9 or above; RMSEA and SRMR should 0.08 or less [40,41]).

## Results

Among the 1985 university students, 938 (47.2%) were Indonesian, 734 (37%) were Thai, and 313 (15.8%) were Taiwanese. Approximately 80% (1588/1985) of all study participants were female, with 82.2% of the participants majoring in medical-related programs (1631/1985) and 80.9% studying at the undergraduate level (1605/1985). The compositions of these aforementioned demographics were significantly different among the 3 groups. More specifically, the Taiwanese student sample comprised more males, the Indonesian student sample comprised more medical students, and the Thai student sample comprised more postgraduates (Table 1).

**Table 1:** Participant characteristics in different groups (N=1985).

Characteristic	Value			*F* test (*df1, df2*)		Chi-square (*df*)		*P* value		Post-hoc comparison	
	Indonesian students (n=938)	Taiwanese students (n=313)	Thai students (n=734)								
Gender, male, n (%)	134 (14.3)	117 (37.4)	146 (19.9)		N/A^a^		78.76 (*2*)		<.001		2>3>1
							
Medical student, yes, n (%)	841 (89.7)	233 (74.4)	557 (75.9)		N/A		565.21 (*2*)		<.001		1>2,3
							
Postgraduate, yes, n (%)	125 (13.3)	37 (11.8)	218 (29.7)		N/A		92.65 (*2*)		<.001		3>1,2
							
Anxiety, mean (SD)	2.33 (0.48)	2.08 (0.42)	2.55 (0.43)		129.19 (*2, 1982*)		N/A		<.001		3>1>2
							
							
Confidence^b^, mean (SD)	2.75 (0.79)	2.37 (0.68)	2.01 (0.86)		172.43 (*2, 1982*)		N/A		<.001		1>2>3
							
Perceived risk^c^, mean (SD)	3.35 (1.16)	3.47 (0.96)	3.15 (1.16)		10.95 (*2, 1982*)		N/A		<.001		1,2>3
							
Support^d^, mean (SD)	4.20 (0.59)	3.89 (0.68)	3.48 (0.70)		259.10 (*2, 1982*)		N/A		<.001		1>2>3
							
Resources^e^, mean (SD)	1.39 (0.50)	1.80 (0.31)	1.31 (0.49)		122.37 (*2, 1982*)		N/A		<.001		2>1>3
							
Internet^f^, mean (SD)	2.73 (0.49)	2.70 (0.49)	2.82 (0.42)		10.59 (*2, 1982*)		N/A		<.001		3>1,2
							
Medical staff^g^, mean (SD)	2.63 (0.58)	2.34 (0.67)	2.37 (0.68)		1.55 (*2, 1982*)		N/A		.21		--
							
Family^h^, mean (SD)	2.52 (0.60)	2.18 (0.59)	2.22 (0.61)		66.52 (*2, 1982*)		N/A		<.001		1>2,3
							

^a^N/A: not applicable.

^b^Confidence: confidence in pandemic control.

^c^Perceived risk: risk perception of susceptibility to COVID-19.

^d^Support: perceived satisfactory support from family, friends, or university.

^e^Resources: sufficiency of resources.

^f^Internet: COVID-19 information received from the internet.

^g^Medical staff: COVID-19 information received from medical staff.

^h^Family: COVID-19 information received from family.

The differences in independent and outcome variables across the 3 student groups are also shown in Table 1. Our results showed that Thai students had the highest levels of anxiety, the lowest levels of confidence in fighting COVID-19, and the least sufficient resources among the 3 student groups. On the other hand, Indonesian students had the highest levels of risk perception of susceptibility to COVID-19 and perceived satisfactory support from different sources among the 3 student groups. Moreover, compared to the other groups, Thai students received more COVID-19 information from the internet, and Indonesian students received more information from medical staff and family (Table 1).

Correlations between independent variables, outcome variables, and controlled variables are presented in Table 2. We found that anxiety was significantly associated with confidence in pandemic control (r=−.08; *P*<.001); risk perception of susceptibility to COVID-19 (r=.07; *P*=.003); sufficiency of resources (r=.08; *P*<.001); and receiving information from the internet (r=.10; *P*<.001), medical staff (r=.11; *P*<.001), and family (r=.08; *P*<.001). Suicidal thoughts were significantly associated with confidence in pandemic control (r=-.28; *P*<.001), perceived satisfactory support (r=-.36; *P*<.001), sufficient resources (r=-.21; *P*<.001), and receiving information from the internet (r=.06; *P*=.006) and family (r=-.12; *P*<.001).

Multigroup SEM showed satisfactory fit indices (CFI=0.92; TLI=0.90; RMSEA=0.051; SRMR=0.053). Regarding the path coefficients for Indonesian students, higher risk perception of susceptibility to COVID-19, greater perceived satisfactory support, and receiving more information from medical staff significantly explained higher levels of anxiety. Less perceived satisfactory support was the only independent variable that significantly explained more frequent suicidal thoughts among the Indonesian students.

Regarding the path coefficients for Taiwanese students, receiving more information from the internet and medical staff significantly explained the higher levels of anxiety observed in this study group. No independent variables significantly explained suicidal thoughts among Taiwanese students.

Finally, regarding the path coefficients for Thai students, only receiving more information from family significantly explained the higher levels of anxiety observed in this study group. Less perceived satisfactory support and receiving less information from medical staff significantly explained the more frequent sadness reported by Thai students (Figure 1).

**Table 2:** Correlation matrix (Pearson r and 2-tailed *P* values) for studied variables (N=1985).

Variable		Gender	Anxiety	Confidence	Perceived risk	Support	Resources	Internet	Medical staff	Family
**Gender**										
	*r*	1	−.08	0.09	−.05	−.07	0.04	−.03	−.02	−.12
	*P* value	—^a^	<.001	<.001	.02)*	.001	-0.06	-0.17	-0.5	<.001
**Anxiety**			—							
	*r*	−.08	1	−.08	0.07	−.03	−.08	0.1	0.11	0.08
	*P* value	<.001	—	<.001	.003	-0.17	<.001	<.001	<.001	<.001
**Confidence^b^**										
	*r*	0.09	−.08	1	0.22	−.35	0.25	0.01	−.10	−.19
	*P* value	<.001	<.001	—	<.001	<.001	<.001	-0.8	<.001	<.001
**Perceived risk^c^**										
	*r*	−.05	0.07	0.22	1	−.01	−.13	−.06	0.1	−.08
	*P* value	.02)*	.003	<.001	—	−0.74	<.001	.008	<.001	<.001)^**^
**Support^d^**										
	*r*	−.07	−.03	−.35	−.01	1	0.22	0.04	0.09	0.27
	*P* value	.001	-0.17	<.001	-0.74	—	<.001	-0.06	<.001	<.001
**Resources^e^**										
	*r*	0.04	−.08	0.25	−.13	0.22	1	0.01	−.08	−.10
	*P* value	-0.06	<.001	<.001	<.001	<.001	—	-0.65	.001	<.001
**Internet^f^**										
	*r*	−.03	0.1	0.01	−.06	0.04	0.01	1	0.14	0.27
	*P* value	-0.17	<.001	-0.8	.008	-0.06	-0.65	—	<.001	<.001
**Medical staff^g^**										
	*r*	−.02	0.11	−.10	0.1	0.09	−.08	0.14	1	0.22
	*P* value	-0.5	<.001	<.001	<.001	<.001	.001	<.001	—	<.001
**Family^h^**										
	*r*	−.12	0.08	−.19	−.08	0.27	−.10	0.27	0.22	1
	*P* value	<.001	<.001	<.001	<.001	<.001	<.001	<.001	<.001	—

^a^Not applicable.

^b^Confidence: confidence in pandemic control.

^c^Perceived risk: risk perception of susceptibility to COVID-19.

^d^Support: perceived satisfactory support from family, friends, or university.

^e^Resources: sufficiency of resources.

^f^Internet: COVID-19 information received from the internet.

^g^Medical staff: COVID-19 information received from medical staff.

^h^Family: COVID-19 information received from family.

## Discussion

### Principal Findings

This study showed that, among the 3 student groups compared, Thai university students had the greatest negative psychological responses (ie, the highest level of anxiety), whereas Taiwanese students had the lowest negative psychological responses. Confidence in pandemic control, sufficiency of resources, and receiving COVID-19 information from the internet and family were all factors associated with both anxiety and suicidal thoughts in the overall study population. Moreover, factors associated with higher levels of psychological responses considerably differed across the 3 countries. For example, less perceived satisfactory support was associated with more suicidal thoughts among Indonesian students and more sadness among Thai students.

Thai students had the highest levels of anxiety among the 3 study groups, which may be attributed to the low confidence they expressed in pandemic control and the lack of resources for fighting COVID-19. Indeed, the correlation results indicated that anxiety and suicidal thoughts were negatively associated with confidence in pandemic control and sufficiency of resources. The main reason for the lowest levels of anxiety and suicidal thoughts among Taiwanese students could be the early reaction by the Taiwan government to control COVID-19 infection [17,42], which substantially decreased disease transmission rate. Consequently, the effects of COVID-19 on daily life were less substantial in Taiwan than in Indonesia and Thailand.

When comparing the various sources of COVID-19 information in relation to anxiety, we found that Taiwanese students were highly affected by COVID-19 information gathered from the internet and from medical staff. This finding was similar to that of another study on the general population in Taiwan, which found that receiving COVID-19 information from the internet was associated with poorer psychological well-being [43,44]. Previous research has indicated that the more an individual gathers internet-based COVID-19 information, the higher is the impact on the individual’s anxiety level, a phenomenon termed “cyberchondria” [43,44]. Seeking health information on the internet has been the most common method to obtain health information even before the COVID-19 pandemic [45]. However, the content and quality of health information available on the internet regarding COVID-19 can vary depending on the region. For example, frequencies of recommendations regarding COVID-19 prevention, such as “wash your hands frequently” or “stay home if you feel unwell,” appearing on the internet were significantly different between Spain and the United States [46].

The proposed model in this study was partially supported because confidence in pandemic control and sufficiency of resources were both associated with more positive psychological responses to the pandemic. This finding indicates that perceived benefits in the HBM is important for university students in overcoming their psychological challenges during the COVID-19 pandemic period. Moreover, the benefits concerning COVID-19 control (ie, confidence in COVID-19 control and resource sufficiency) appeared to be more important than the benefits of others’ emotional support (ie, perceived support). Moreover, perceived satisfactory support appeared to be an important factor in our study given the contrasting findings concerning anxiety and suicidal thoughts in some cultures. Lower perceived satisfactory support was associated with greater suicidal thoughts among Indonesian students and more sadness among Thai students, but more perceived support was associated with higher anxiety among Indonesian students. Perceived support may prevent individuals from having suicidal thoughts or sadness, but it may result in increased anxiety due to sharing of COVID-19 information repeatedly in a smaller social network. Moreover, information on the internet may even contain misinformation concerning COVID-19 [47]. The general public needs to know where on the internet to seek accurate information related to COVID-19 and not to constantly check for new information, as this could negatively affect their psychological health. However, individuals are still encouraged to seek information because accurate information can help them to engage in appropriate preventive behaviors. Therefore, we recommend that the public should seek information moderately. In some cultures, such as among the Thai university students in this study, receiving more COVID-19 information from medical staff was associated with less sadness. This finding suggests that a more reliable source of COVID-19 information may subsequently reduce suicidal thoughts among Thai students, given that feeling sad or experiencing a low mood is one of the depressive symptoms that could lead to suicide. Future studies should also analyze the content, frequency, and the various types of COVID-19 information available online that may be directly associated with anxiety. This knowledge could be used to promote additional sources on COVID-19 information for the general population such that they can find reliable and accurate information.

The amount of information may be amplified due to echo chamber effects, wherein information is disseminated among those who know each other very well, and individuals believe and trust in the information shared because they are very familiar with each other [48]. Thus, if misinformation was spread and exacerbated in their networks, it is likely that their anxiety levels will increase. However, if individuals can obtain accurate COVID-19 information through their close social network, it could lead to a reduction in suicidal thoughts. The findings of our study also highlight that the risks of university students having suicidal thoughts during the COVID-19 pandemic should not be overlooked.

Based on the findings of this study, there are several implications. First, these findings suggest there is a need for health care providers to help improve the psychological health of university students by providing them with reliable information to increase their confidence in COVID-19 pandemic control and provide sufficient resources in dealing with the resulting psychological impact. More specifically, psychoeducation to assist university students in understanding how the city and university are controlling and minimizing the spread of COVID-19 may be one method for improving students’ confidence in COVID-19 control. Additionally, programs highlighting preventive COVID-19 behaviors may also increase students’ confidence in controlling COVID-19 at an individual level. Second, health care providers need to provide sufficient PPE, accurate COVID-19 information, monetary and medical resources, and psychological support to students. This would help students perceive the benefits of these resources in minimizing the impact of COVID-19 and decreasing their psychological distress. Third, health care providers should encourage students to obtain COVID-19 information from reputable and trusted sources, such as from medical staff, rather than from the internet and social media, as misinformation on the internet may be difficult to identify and may have a negative impact on the students’ psychological health. In addition, health care providers also need to be proactive in correcting misinformation spread among university students.

### Study Limitations

There are some limitations to this study. First, the majority of the university students in the present study were female majoring in medical-related programs. Therefore, the generalizability of the findings is restricted. Future studies with university students majoring in other programs (eg, social science and engineering) are therefore needed. Moreover, the present findings are arguably biased because, in general, medical professionals are subject to experiencing more psychological impacts due to the nature of their work requiring them to be at the frontline, especially during the ongoing COVID-19 pandemic. Second, although the present study recruited participants from different East Asian countries, the sample representativeness is restricted due to the sample sizes being different in the 3 countries studied. More specifically, the Taiwanese sample was relatively smaller (15.8%) than the other two samples. Therefore, the country comparisons are limited. Third, all the measures were assessed using self-reports received via a web-based survey. The biases of social desirability and common method variance may therefore influence these results. Fourth, confidence in pandemic control was assessed by combining 3 different domains (ie, personal, university, and city). Given that confidence in oneself can considerably differ from that in how a university or city handles a public health emergency situation, the confidence in pandemic control examined in the present study was not specific. Therefore, future studies should consider separating the confidence in different domains and examining the effects of each type of confidence. Finally, the response scales used in the present study were different for different variables (eg, the items concerning confidence in pandemic control used a 5-point scale, and items concerning risk perception of susceptibility to COVID-19 used a 7-point scale). The use of different response scales may have affected instrument reliability and validity. However, this may not be a serious problem because instruments using different response scales within the same measure (eg, Short-Form 36) have been shown to have good psychometric properties [49].

### Conclusions

In conclusion, this study demonstrated that university students from different countries had different levels of psychological responses relating to the COVID-19 pandemic. Moreover, receiving more COVID-19 information appears to increase the anxiety levels among university students, but not in relation to suicidal thoughts. Receiving less satisfactory support was found to be associated with more suicidal thoughts among Indonesian students and greater sadness among Thai students. Therefore, health care providers need to establish a good support system for university students to get through the current pandemic.

## Abbreviations

ANOVA: analysis of variance
CFI: comparative fit index
HBM: health belief model
PMT: protection motivation theory
PPE: Personal protective equipment
RMSEA: root mean square error of approximation
SEM: structural equation modeling
SRMR: standardized root mean square residual
STAI: State-Trait Anxiety Inventory
TLI: Tucker-Lewis index
